# Effectiveness of a Glo Germ-based assessment and educational intervention to improve hand hygiene compliance among hospital cleaning staff

**DOI:** 10.3389/fpubh.2025.1694440

**Published:** 2026-01-28

**Authors:** Yachen Chu, Qiu Yao, Fengling Tan, Ying Li, Xuling Pan

**Affiliations:** 1 Department of Pain, The Second Affiliated Hospital of Soochow University, Suzhou, China; 2 Department of Urology, The Second Affiliated Hospital of Soochow University, Suzhou, China; 3 Department of Infection Control, The Second Affiliated Hospital of Soochow University, Suzhou, China; 4 Department of Obstetrics and Gynecology, The Second Affiliated Hospital of Soochow University, Suzhou, China

**Keywords:** cleaning staff, educational intervention, Glo Germ, hand hygiene, pain

## Abstract

**Objective:**

This study aimed to assess the effectiveness of a Glo Germ-based method in evaluating hand hygiene (HH) compliance among hospital cleaning staff and examine the impact of a corresponding educational intervention on improving HH practices. Glo Germ, a fluorescent simulation agent, provides a visually intuitive tool for HH assessment, especially for staff with limited medical backgrounds; residual fluorescence under UV light indicates inadequate cleaning. Eighty cleaning staff (54 females, 26 males; mean age 48.5 ± 6.4 years, ≥1 year of experience, prior HH training) were included. A questionnaire assessed HH knowledge (indications, techniques, duration), and the 45-min educational intervention involved demonstration and hands-on practice. No long-term follow-up was conducted.

**Methods:**

Baseline HH efficacy was assessed via Glo Germ and a structured questionnaire. Targeted educational interventions were implemented based on baseline results, followed by reassessment using the same methods. Pre- and post-intervention handwashing pass rates and knowledge scores were compared.

**Results:**

Pre-intervention, 27/80 (33.75%) participants showed residual fluorescence (most frequent in interdigital spaces: 13.75%, fingertips: 12.50%, dorsal fingers 2–5: 11.25%). Post-intervention, non-compliance dropped to 7/80 (8.75%, *p* < 0.05). Knowledge pass rates for HH indications, techniques, and duration also significantly increased (*p* < 0.05).

**Conclusion:**

Glo Germ is a rapid, effective tool for HH assessment among hospital cleaning staff. Combined with targeted educational interventions, it significantly enhances HH practices and knowledge.

## 1 Introduction

Cleaning staff are responsible for routine environmental disinfection in clinical and patient areas, leading to frequent contact with potentially contaminated surfaces and fomites, which places them at risk of becoming vectors for pathogen transmission. Standard Precautions, particularly hand hygiene (HH), are recognized as one of the most essential, straightforward, effective, and cost-efficient measures for preventing and controlling healthcare-associated infections (HAIs), thereby safeguarding both patients and healthcare personnel (1–3). Although the WHO Multi-modal Hand Hygiene Improvement Strategy has shown progressive improvement in implementation scores, sustained efforts are still needed to achieve optimal compliance (1). Recent observational studies further underscore the importance of monitoring and promoting HH compliance among healthcare workers through direct observation and objective measures such as adenosine triphosphate assays, highlighting ongoing challenges and opportunities in diverse clinical settings (4–6). Though HH compliance among healthcare professionals is highly variable (range 9–100%), most point-prevalence surveys report 60–70% (7). In contrast, hospital cleaning staff usually demonstrate lower HH compliance (often < 40%) and poorer knowledge scores compared with clinical staff (8, 9). This disparity has been attributed to factors such as lack of formal medical training (10), high staff turnover (11), and limited opportunities for ongoing education and compliance monitoring. These challenges render infection prevention and control among cleaning staff a particularly complex aspect of hospital management.

Currently, HH compliance is assessed by four main strategies: (1) direct human observation—considered the gold standard but subject to Hawthorne bias and high labour cost (12); (2) electronic/automated systems that record dispenser activations or use wearable sensors—providing continuous data but requiring substantial infrastructure and maintenance (8, 9); (3) fluorescent-simulation agents such as Glo Germ that allow immediate visual feedback under UV light, are inexpensive, and can be integrated directly into training (11); (4) Additionally, validated self-reported scales (e.g., Lam 2014; Lommi 2023) offer a low-cost alternative for multi-site comparison, although they are not considered a gold standard (13, 14). For cleaning staff who have limited medical background and intermittent access to electronic systems, the third option offers a pragmatic, low-cost solution that simultaneously serves as an educational tool. We therefore selected oil-based Glo Germ for both baseline assessment and the subsequent targeted intervention.

To address the need for a rapid, visually engaging and demonstrable method for assessing HH performance and enhancing compliance with standardized practices, an oil-based Glo Germ ultraviolet fluorescent agent was employed in this study to simulate microbial contamination. This approach was used to evaluate HH efficacy among cleaning staff and to implement targeted interventions aimed at improving HH practices. While numerous studies have focused on HH compliance among clinical staff, hospital cleaning staff—who are equally exposed to pathogens—remain underrepresented in research and intervention efforts. This study addresses this gap by evaluating and improving HH practices specifically among cleaning personnel. The primary objective of this study was to evaluate the immediate impact of a Glo Germ-based educational intervention on handwashing technique among hospital cleaning staff. Secondary objectives included assessing its effect on HH-related knowledge and identifying the most commonly missed hand areas before and after the intervention.

## 2 Methods

### 2.1 Study participants

This was a single-group pre--post interventional study conducted in June 2025. The study was conducted at The Second Affiliated Hospital of Soochow University, a tertiary hospital with approximately 3,000 beds and over 236 cleaning staff members. In June 2025, eighty hospital cleaning staff members from the study institution were enrolled, comprising 26 males and 54 females, aged between 39 and 65 years (mean age: 48.5 ± 6.4 years). Among them, 58 (72.5%) were designated to work mainly in clinical areas such as wards, clinics, and operation theatres, while 22 (27.5%) worked in non-clinical areas including the main lobby, lifts, and administration offices. Among 74 respondents, 46 (62.2%) had completed primary school, 25 (33.8%) secondary school, and 3 (4.1%) technical or junior college education. Participants were recruited through convenience sampling via departmental supervisors. All eligible cleaning staff were invited to participate voluntarily. This method may introduce selection bias, which is acknowledged as a limitation. No formal sample size calculation or power analysis was conducted prior to the study. The sample size was determined based on practical availability. This limitation is acknowledged, and future studies are encouraged to include power analyses.

### 2.2 Study methods

Pre-intervention handwashing efficacy was evaluated using the oil-based Glo Germ method. Glo Germ is a highly fluorescent melamine copolymer, approximately 5 μm in diameter, that is safe, non-toxic, and invisible under natural light. An oil-based Glo Germ preparation was applied to the palms and evenly distributed to ensure coverage of the palmar and dorsal surfaces of both hands, thumbs, fingers (palmar and dorsal surfaces of digits 2–5), interdigital spaces, fingertips, and wrists. Under dim lighting, ultraviolet light examination confirmed fluorescence distribution across all designated areas. The educational intervention lasted approximately 45 min and was delivered by a certified infection control nurse with over 10 years of experience. It included a brief lecture (10 min), a live demonstration of the WHO 7-step handwashing technique (10 min), and hands-on practice with Glo Germ under UV light (25 min).

Dosage, frequency and duration: each participant received a single 45-min session (no booster). The 10-min mini-lecture duration was chosen because Öncü et al. (15) achieved significant knowledge improvement among cleaners with≤9 years schooling using 7–15 min lectures plus demonstration.

Each participant completed a structured questionnaire assessing knowledge of three key HH components: handwashing indications (before touching a patient, before clean/aseptic procedure, after body fluid exposure risk, after touching a patient, and after touching patient surroundings), scrubbing techniques (palmar and dorsalsurfaces of both hands, thumbs, palmar and dorsal surfaces of digits 2–5, interdigital spaces, fingertips, and wrists), and required scrubbing duration (40–60 s for handwashing with soap and water). All sessions followed a standardized protocol to ensure consistency across participants. The questionnaire was constructed from the 2009 WHO Hand Hygiene Technical Reference Manual (pp.30–35) and the local protocol ‘SOP-IC-017 Hand Hygiene for Environmental Services’(rev. 2023-03-01). Content validity was examined by three infection-control nurses (average 12 years IC experience); clarity index ≥ 0.80 was required for item retention. The educational intervention was conducted immediately after the baseline assessment and lasted approximately 45 min.

A practical approach to assess hand hygiene technique and monitor its compliance is by using a fluorescent gel (16). It was reviewed by infection control specialists for content accuracy. However, formal validation or reliability testing (e.g., Cronbach’s alpha) was not conducted, which is acknowledged as a limitation.

Based on the initial Glo Germ residue patterns and questionnaire scores, standardized HH training was conducted, incorporating targeted interventions to address identified deficiencies in handwashing performance while providing specific guidance for improvement.

Following training, handwashing efficacy was reassessed using the same Glo Germ protocol, and the knowledge questionnaire was re-administered. Statistical analyses compared pre- and post-intervention data, including the number of participants with inadequate scrubbing in specific hand regions, the total number of inadequate handwashing cases, and HH knowledge assessment scores.

The educational intervention was targeted based on the most common hand regions with residual fluorescence (e.g., interdigital spaces, fingertips) and the lowest-scoring knowledge domains (e.g., handwashing duration and technique). It lasted approximately 45 min and was delivered by a certified infection control nurse with over 10 years of experience. The session included a 10- min lecture on the importance of hand hygiene and common mistakes; a 10- min live demonstration of the WHO 7-step handwashing technique using Glo Germ under UV light; and a 25-min hands-on practice session where participants applied Glo Germ, washed their hands, and received immediate UV feedback.

No long-term follow-up was conducted due to logistical constraints. Future studies are recommended to assess sustainability of improvements at 1–3 months post-intervention.

### 2.3 Rationale for choosing oil-based Glo germ

The principal advantages of oil-based Glo Germ for HH assessment and intervention in this occupational group are as follows:

(1) Visual demonstration: The method enables participants to visualize simulated “pathogens,” making it particularly suitable for cleaning staff with limited formal medical education.
(2) Real-time feedback: Unlike traditional hand sampling followed by microbial culture, which requires delayed evaluation through colony counts, Glo Germ provides immediate feedback on handwashing effectiveness. The observed distribution of residual fluorescence highlights specific anatomical regions that require improved scrubbing.
(3) Integrated assessment-intervention-reassessment process: In contrast to traditional Plan–Do–Check–Act (PDCA) management cycles, which involve multiple steps and are time-intensive, this approach allows assessment, targeted intervention, and reassessment to be completed within a single training session. This streamlines management procedures, reduces time demands, and improves human resource efficiency.
(4) Ease of implementation: The procedure requires only oil-based Glo Germ fluorescent agent, ultraviolet lighting, and standard HH facilities. The necessary materials and equipment are inexpensive and readily available.

### 2.4 Statistical analysis

Data were analyzed using SPSS software, version 19.0 (IBM Corp., Armonk, NY, USA). Categorical variables were compared using the chi-square test, with statistical significance set at *p* < 0.05. Categorical variables (e.g., pass/fail rates for handwashing) were compared using McNemar’s test. Continuous variables (e.g., knowledge scores) were analyzed using paired *t*-tests or Wilcoxon signed-rank tests, depending on normality distribution. A *p*-value < 0.05 was considered statistically significant. All analyses were performed using SPSS version 19.0.

## 3 Results

### 3.1 Inadequate hand scrubbing and overall handwashing performance before and after intervention

**Figure 1:**
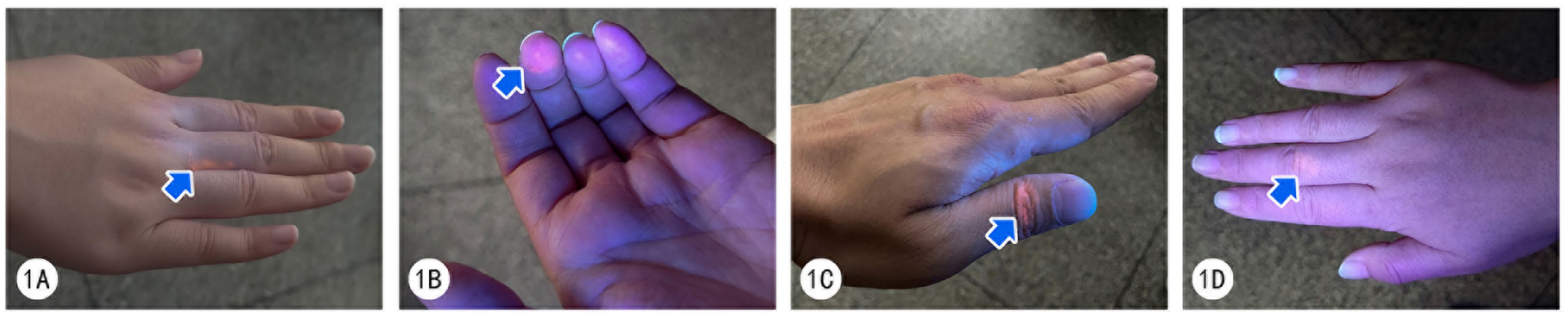
Visualization of Glo Germ residue under ultraviolet light. **(A)** Interdigital space between the third and fourth fingers. **(B)** Fingertip of the third finger. **(C)** Thumb. **(D)** Dorsal surface of the middle finger.

Residual Glo Germ fluorescence after handwashing was visualized under ultraviolet light (Figure 1). Table 1 summarizes the number of participants with fluorescent residue in specific hand regions, including palms, dorsal surfaces, thumbs, palmar and dorsal surfaces of fingers 2–5, interdigital spaces, fingertips, and wrists, as well as overall inadequate handwashing performance, before and after the intervention.

**Table 1:** Comparison of the number and percentage of participants with Glo Germ residue in each hand region and overall inadequate handwashing performance before and after the intervention.

Region		Palms	Dorsal surfaces	Thumbs	Palmar surfaces of fingers 2–5	Dorsal surfaces of fingers 2–5	Interdigital spaces	Fingertips	Wrists	Inadequate handwashing (*n*)
Before intervention	Number (*n*)	2	2	7	3	9	11	10	7	27
	Percentage (%)	2.50	2.50	8.75	3.75	11.25	13.75	12.50	8.74	33.75
After intervention	Number (*n*)	0	0	1	0	1	3	2	0	7
	Percentage (%)	0	0	1.25	0	1.25	3.75	2.50	0	8.75
*x*^2^ value		14.939								
*p* value		0.000								

**Figure 2:**
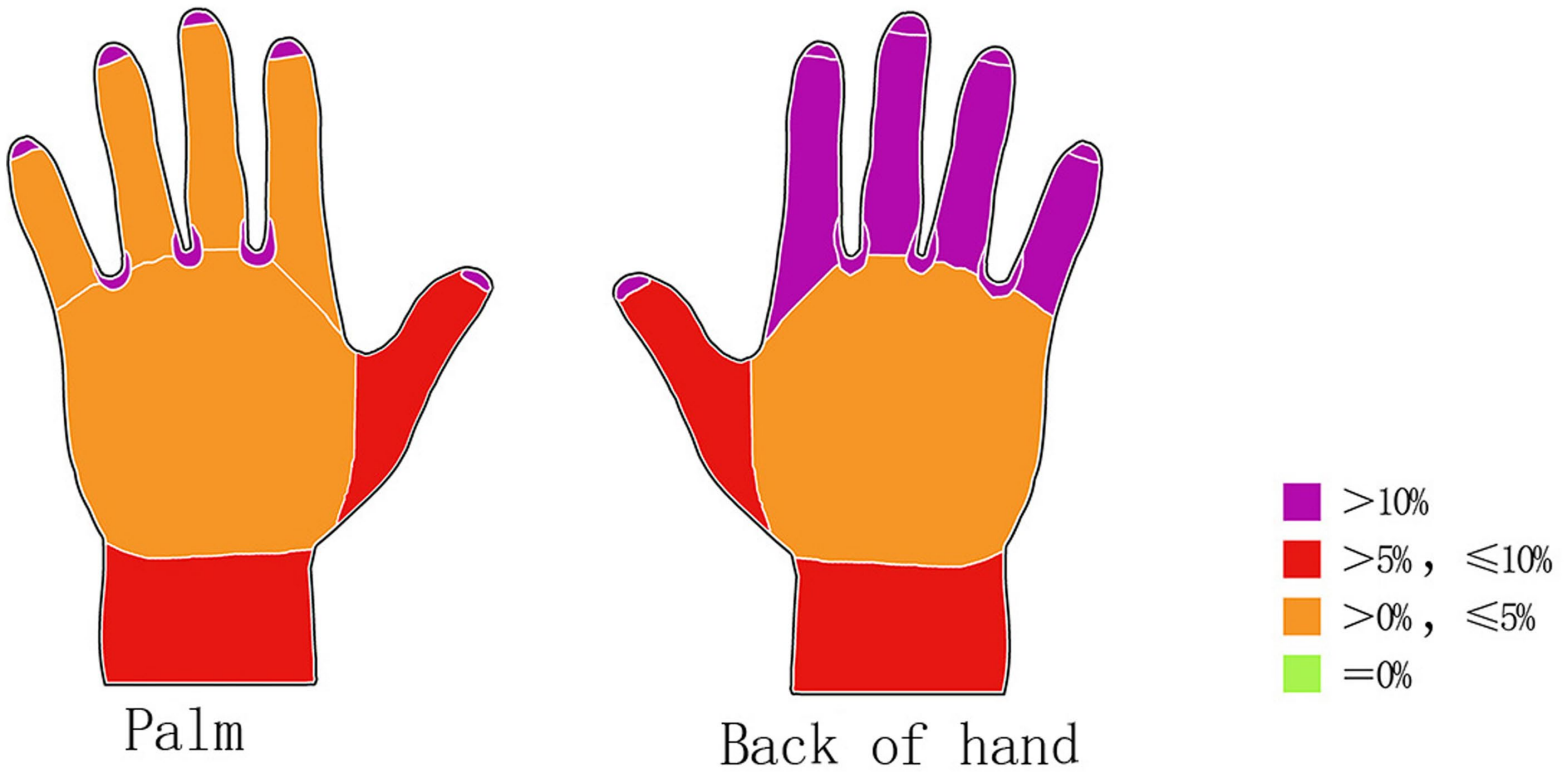
Distribution of Glo Germ residue by anatomical region before the intervention (palmar surface; dorsal surface).

**Figure 3:**
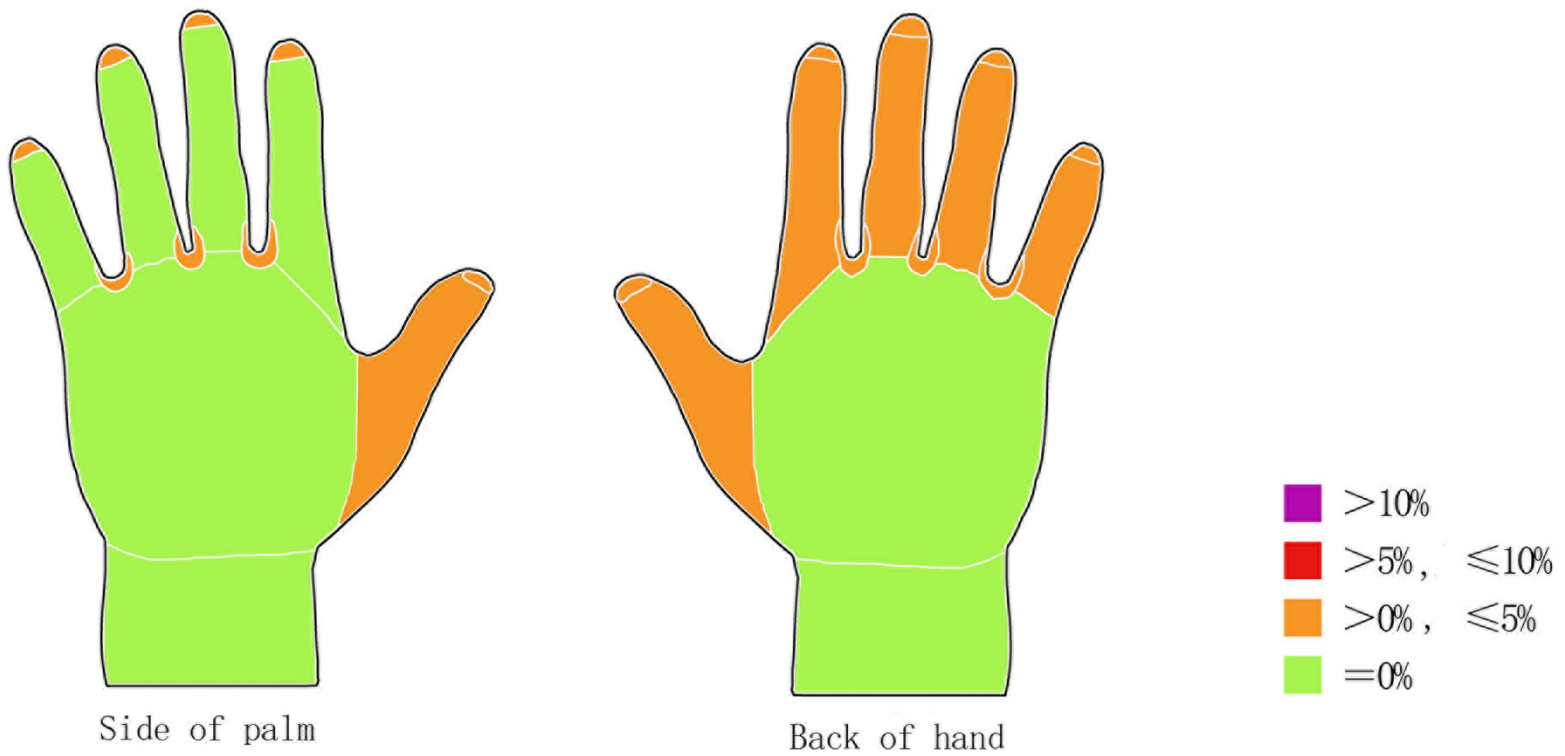
Distribution of Glo Germ residue by anatomical region after the intervention.

Among the 80 cleaning staff members, 27 (33.75%) demonstrated inadequate handwashing performance during the initial assessment, as indicated by the presence of Glo Germ residue. Fluorescent residue was most frequently detected in the interdigital spaces (11 participants, 13.75%), followed by fingertips (10 participants, 12.50%) and the dorsal surfaces of fingers 2–5 (9 participants, 11.25%). These areas represented the most common sites of insufficient scrubbing and incomplete cleaning (Figure 2). Figure 2 shows that prior to intervention, the most frequently missed areas were the interdigital spaces (13.75%), fingertips (12.50%), and dorsal surfaces of fingers 2–5 (11.25%). Post-intervention (Figure 3), residual fluorescence in these areas was reduced to 3.75, 2.50, and 2.50%, respectively.”

Following the targeted training intervention, the number of participants with fluorescent residue in each region was reduced. The proportion of participants with overall inadequate handwashing performance declined to 7 (8.75%), a statistically significant improvement (McNemar’s test, *x*^2^ = 12.8, df = 1, Cohen’s *h* = 0.62), representing a statistically significant improvement over pre-intervention results (*p* < 0.01). Post-intervention residue distribution is shown in Figure 3. The high frequency of residual fluorescence in interdigital spaces and fingertips is consistent with prior studies, likely due to inadequate attention to these anatomically complex and less accessible areas during routine handwashing. Post-intervention, the interdigital spaces remained the most challenging area, with 3 participants (3.75%) still showing residue. Future training should emphasize repeated scrubbing of these areas.

### 3.2 HH knowledge assessment before and after intervention

Prior to the intervention, the number of participants achieving passing scores in the knowledge assessments for HH indications, scrubbing techniques, and scrubbing duration was 62 (77.50%), 67 (83.75%), and 69 (86.25%), respectively. Pass rates increased significantly for all three knowledge domains (McNemar’s test, *x*^2^ ≥ 8.1, df = 1, *p* < 0.01 for all comparisons). Following the training intervention, these numbers increased to 76 (95.00%), 78 (97.50%), and 80 (100%), respectively. All post-intervention improvements were statistically significant compared with pre-intervention results (*p* < 0.01) (Table 2). Table 2 shows that pre-intervention pass rates were lowest for handwashing indications (77.5%), followed by scrubbing technique (83.75%) and duration (86.25%). Post-intervention, all domains reached ≥95% pass rates. Mean knowledge scores increased from 78.4 ± 9.2 pre-intervention to 92.6 ± 5.8 post-intervention (paired *t*-test, *t* = 11.3, df = 79, *p* < 0.001). No statistically significant gender differences were observed in baseline knowledge scores (females 79.1 ± 8.7 vs. males 77.9 ± 10.2; *p* = 0.58) or post-intervention improvement (Δscore +14.0 ± 9.1 vs. + 13.6 ± 8.4; *p* = 0.86). The reduction of inadequate hand-washing was likewise comparable between females and males (*p* = 0.78). Median scores improved from 80 (IQR: 72–85) to 94 (IQR: 90–98).

**Table 2:** Comparison of hand hygiene knowledge assessment pass rates before and after the intervention.

Assessment item	Before intervention		After intervention		*x*^2^ value	*p* value
	Pass (*n*)	Pass rate (%)	Pass (*n*)	Pass rate (%)		
Hand washing indications	62	77.50	76	95.00	10.329	0.001
Scrubbing techniques	67	83.75	78	97.50	8.901	0.003
Scrubbing duration	69	86.25	80	100.00	11.812	0.001

### 3.3 Correlation analysis between knowledge enhancement and handwashing effect

Subgroup analyses showed no statistically significant differences in improvement by sex or age group (all *p* > 0.05), although younger participants (≤45 years) showed slightly greater improvement in knowledge scores. A moderate positive correlation was observed between improvement in knowledge score and reduction in residual fluorescence (Pearson’s *r* = 0.41, *p* < 0.001), suggesting that participants with greater knowledge gains also showed better handwashing outcomes.

### 3.4 Impact of working places and educational level on HH knowledge and compliance content

To further explore potential factors influencing HH outcomes, we analyzed the impact of working places (clinical vs. non-clinical areas) and educational level on baseline knowledge scores and post-intervention improvements.

Regarding working places, 58 participants worked in clinical areas (wards, clinics, operating theatres) and 22 in non-clinical areas (lobby, lifts, administration offices). Statistical analysis showed no statistically significant differences between the two groups in baseline HH knowledge scores (clinical areas: 79.1 ± 8.9; non-clinical areas: 76.8 ± 9.5; independent samples *t*-test, *t* = 1.03, df = 78, *p* = 0.305), post-intervention knowledge improvement (clinical areas: 13.8 ± 8.7; non-clinical areas: 14.2 ± 9.3; independent samples *t*-test, *t* = 0.18, df = 78, *p* = 0.857), or reduction in inadequate handwashing rates (clinical areas: 26.7% reduction from 36.2 to 9.5%; non-clinical areas: 23.1% reduction from 27.3 to 4.2%; *x*^2^ = 0.32, df = 1, *p* = 0.571).

For educational level, participants were categorized into lower education (primary school, *n* = 46) and higher education (secondary school + technical/junior college, *n* = 28) groups. The higher education group had significantly higher baseline HH knowledge scores compared to the lower education group (81.9 ± 8.2 vs. 76.3 ± 9.4; independent samples *t*-test, *t* = 2.41, df = 72, *p* = 0.018). However, there were no significant differences between the two groups in post-intervention knowledge improvement (lower education: 13.5 ± 8.9; higher education: 14.3 ± 8.6; independent samples *t*-test, *t* = 0.36, df = 72, *p* = 0.721) or reduction in inadequate handwashing rates (lower education: 24.8% reduction from 34.8 to 10.0%; higher education: 27.9% reduction from 32.1 to 4.2%; *x*^2^ = 0.15, df = 1, *p* = 0.698).

These results indicate that the Glo Germ-based educational intervention is equally effective across different working places and educational levels of cleaning staff.

## 4 Discussion

HH is a well-established and effective measure for reducing healthcare-associated infections (4). Although physicians and nurses usually achieve higher baseline knowledge scores than cleaners, their observed HH compliance is still sub-optimal (median 60–70%), indicating that both groups require continuous reinforcement (2, 7, 12). Although cleaning staff are not directly involved in patient care, their frequent contact with patient environments places them at risk of serving as vectors for HAI transmission. Evidence from domestic and international studies (8, 9, 12) indicates that cleaning staff often have limited HH knowledge, insufficient adherence to procedural standards, and low compliance rates. However, in the present study, prior to intervention, 77.5% of the 80 participating cleaning staff passed the assessment in hand hygiene indications and more than 80% of them got a pass in HH knowledge assessment in scrubbing technique and duration. This may be explained as all participants had completed their job- specific orientation training, with a minimum of one year of hospital cleaning experience, and participated in at least one standardized HH training session before. Despite this, only 66.25% of them demonstrated adequate handwashing performance during the initial assessment. All these findings highlighted the necessity of improving HH knowledge and compliance among this occupational group to support effective HAI prevention. Targeted educational intervention among hospital cleaning staff could increase HH knowledge and improvhandwashing performance.

Multiple factors contribute to suboptimal HH performance among cleaning staff in this study: (1) Lower educational attainment limits risk perception and technique comprehension (11); (2) Insufficient administrative priority results in poor training supervision and performance feedback (8); (3) Traditional verbal-only training lacks visual feedback and actionable improvement strategies (9); (4) Low compensation and high staff turnover reduce sustained engagement in compliance initiatives (17); (5) Inadequate facilities and supplies (e.g., inaccessible sinks or empty dispensers) further hinder compliance (18).

For example, a 2019 study by Sendall et al. among Australian hospital cleaners reported baseline HH compliance below 40%, similar to our findings (9). Another study by Xiao et al. (8) in China also identified knowledge gaps and poor technique among cleaning staff, reinforcing the need for targeted interventions like ours.

Our supplementary analyses explored the impacts of working places (clinical vs. non-clinical) and educational level on HH outcomes—factors previously linked to infection control (9, 10). Notably, working place did not significantly influence baseline HH knowledge or the intervention’ s effectiveness, likely due to uniform mandatory HH training for all cleaning staff during orientation, which ensures a basic level of HH awareness regardless of work area. Regarding educational level, higher attainment correlated with better baseline HH knowledge consistent with Xiao et al. (8) and Chen et al. (10), but the Glo Germ-based intervention yielded comparable improvements in knowledge and handwashing compliance across educational groups. This reflects the intervention’s visual, hands-on design, which overcomes educational barriers (e.g., limited processing of verbal/written instructions) via immediate, intuitive feedback—particularly valuable for cleaning staff with diverse educational backgrounds. These findings enhance the intervention’s generalizability, confirming its effectiveness for staff in both clinical/non-clinical settings and across varying educational levels.

Persistent fluorescence in interdigital spaces and fingertips reflects both anatomical complexity and habitual neglect. These areas are harder to reach and often receive less friction during washing. Future interventions could include repeated guided practice with UV feedback, visual reminders, or even wearable prompts to encourage thorough scrubbing.

Comprehensive administrative interventions, including HH education and promotion, infrastructure improvement, and robust monitoring and feedback systems, have been shown to enhance compliance and pass rates (10, 11). However, no consensus exists regarding the establishment of standardized training, assessment, and evaluation systems specifically designed for the needs and characteristics of cleaning staff.

When Glo Germ exposed to ultraviolet illumination, it emits bright fluorescence, enabling the visual simulation of microbial contamination (18). It is commercially available in powder, oil-based, and gel formulations. Both domestic and international studies have applied in various medical simulations, including endotracheal intubation, donning and doffing personal protective equipment, and surgical operations to model microbial transmission (3, 10, 15, 16, 18). These investigations have demonstrated that Glo Germ is an effective, rapid, and intuitive microbial surrogate. Given the observed decline in similar programs elsewhere, it is unlikely that a one-time intervention will sustain long-term behavior change. Periodic reinforcement sessions, ideally integrated into routine training cycles, are recommended.

Our findings may be generalizable to similar tertiary hospitals in urban China, particularly those with comparable cleaning staff demographics. However, caution is needed when extrapolating to rural settings, private hospitals, or staff with higher baseline education or training. Oil-based Glo Germ is predominantly utilized for HH training and the evaluation of handwashing effectiveness. In a study by Öncü et al., oil-based Glo Germ was employed to simulate microorganisms during the instruction of the seven-step handwashing techniques to children, resulting in improved handwashing initiative, enhanced hygiene habits, and increased accuracy and efficacy in handwashing performance (15).

To address existing HH deficiencies among hospital cleaning staff, oil-based Glo Germ was utilized for both assessment and training. The intervention resulted in improved HH pass rates, and its visualization capability enhanced comprehension and retention of HH-related knowledge. Long-term follow-up is essential to determine whether observed improvements persist over time. Future studies should assess compliance at 1, 3, and 6 months post-intervention. Hospital administrators should consider integrating Glo Germ-based training into existing infection control programs. Embedding quarterly retraining sessions and combining visual feedback with routine compliance audits could enhance long-term adherence and accountability.

However, this study has certain limitations. This study has several limitations. First, the absence of blinding may have introduced observer bias during Glo Germ assessments. Second, the lack of long-term follow-up limits conclusions about sustainability. Third, the questionnaire was not formally validated, and self-report bias may have influenced responses. Fourth, the single-group design precludes causal inference. Finally, convenience sampling may limit generalizability. Glo Germ functions as a microbial simulation agent rather than a biological marker of microorganisms. Consequently, the detection of fluorescent residue under ultraviolet light is an indirect measure of handwashing thoroughness, serving as a rapid method for evaluating HH effectiveness (19). The reliability of this approach should be confirmed through large-sample comparative analyses between fluorescent residue patterns and microbial colony counts obtained from hand cultures. Furthermore, the relatively small sample in the present study limits the generalizability of the findings, underscoring the need for validation in studies with larger and more diverse participant groups.

## 5 Conclusion

This study demonstrated that a Glo Germ-based educational intervention significantly improved hand hygiene technique and knowledge among hospital cleaning staff in a single 45-min session. This intervention can be easily integrated into routine orientation or quarterly training sessions, requiring minimal resources and time. Its visual feedback mechanism enhances engagement and retention, making it ideal for low-literacy or non-clinical populations. Although conducted in a single hospital, the simplicity and low cost of this approach make it adaptable to other healthcare settings with similar staff profiles and resource constraints. Future research should include multi-center randomized controlled trials with long-term follow-up to assess sustainability. Additionally, linking hand hygiene improvements to reductions in healthcare-associated infections would strengthen the evidence base for this intervention. These findings support the integration of Glo Germ-based training into routine hospital infection control policies, particularly for non-clinical staff. The low-cost, visual nature of the intervention makes it scalable across departments and suitable for quarterly reinforcement sessions. By improving hand hygiene compliance among cleaning staff, hospitals can reduce environmental pathogen transmission, enhance overall infection prevention efforts, and meet accreditation standards more effectively.

Compared with clinical staff, cleaning staff face distinct barriers: lower health-literacy, fewer training opportunities, high turnover and low supervision. Future interventions should address these structural factors through simplified visual tools, periodic reinforcement and supervisory feedback.

## Data Availability

The original contributions presented in the study are included in the article/supplementary material, further inquiries can be directed to the corresponding author.
